# Alleviating Plant Density and Salinity Stress in *Moringa oleifera* Using Arbuscular Mycorrhizal Fungi: A Review

**DOI:** 10.3390/jof11040328

**Published:** 2025-04-21

**Authors:** Tshepiso Khoza, Absalom Masenya, Nokuthula Khanyile, Standford Thosago

**Affiliations:** 1 School of Agriculture, University of Mpumalanga, Private Bag X11283, Mbombela 1200, South Africa; 201943298@ump.ac.za (T.K.);; 2 School of Chemical and Physical Sciences, University of Mpumalanga, Private Bag X11283, Mbombela 1200, South Africa; khanyile.peaceful@gmail.com

**Keywords:** arbuscular mycorrhizal fungi, common mycorrhizal network (CMN), photosynthetically active radiation (PAR), phytochemicals, reactive oxygen species (ROS)

## Abstract

*Moringa oleifera* (LAM) is a multipurpose tree species with extensive pharmacological and ethnomedicinal properties. Production of important medicinal plants is facing decline under changing climatic conditions, which brings along exacerbated abiotic stresses like salinity and intraspecific competition, particularly high planting densities. Increasing plant density is seen as a strategy to increase production; however, the intraspecific competition and a lack of arable land limit productivity. Salinity has been estimated to harm approximately six percent of the Earth’s landmass. This leads to a loss of over 20% of agricultural output annually. These stressors can significantly curtail moringa’s growth and yield potential. Literature designates that Arbuscular Mycorrhizal Fungi (AMF), ubiquitous soil microorganisms forming symbiotic associations with plant roots, offer a promising avenue for mitigating these stresses. This narrative review aims to investigate the utilization of AMF to alleviate the detrimental effects of salinity and high planting density on *Moringa oleifera*. The different adaptive strategies *M. oleifera* undergoes to mitigate both stressors are explored. The review found that AMF inoculation enhances plant tolerance to these stressors by improving nutrient acquisition, water relations, and activating stress response mechanisms. By facilitating improved nutrient and water absorption, AMF enhance root architecture, modulate ROS scavenging mechanisms, and promote optimal biomass allocation, ensuring better survival in high-density plantings. Furthermore, AMF-mediated stress alleviation is linked to enhanced physiological efficiency, including increased chlorophyll content, root–shoot biomass balance, and ion homeostasis. This review is important because it could provide insights into a sustainable, natural solution for improving the resilience of *Moringa oleifera* under adverse environmental conditions, with potential applications in global agriculture and food security. Future research should prioritize identifying and characterizing moringa-specific AMF species and evaluate the long-term efficacy, feasibility, and economic viability of AMF application in real-world moringa cultivation systems to fully harness the potential of AMF in moringa cultivation.

## 1. Introduction

*Moringa oleifera* (Lam.), native to the sub-Himalayan regions of the Indian subcontinent, is one of 13 known species in the Moringaceae family [1]. These fast-growing deciduous trees are cultivated across diverse regions, including India, Sudan, Southern Africa, the Pacific Islands, the Caribbean, and South America [2]. Moringa is highly valued for its extensive phytochemical content, offering pharmacological benefits such as anti-infertility, anti-ulcer, anticancer, hepatoprotective, antimicrobial, and antidiabetic properties [3]. This medicinal plant has phytochemicals, biologically active yet nutritionally inert compounds that protect plants from microbial threats and are synthesized via primary and secondary metabolic pathways [4]. India dominates the global production and trade of *M. oleifera*, offering a variety of products like canned goods, fresh fruits, seeds, and leaf powders [5]. The country’s annual output of tender pods ranges between 1.1 million—1.3 million tons [5,6]. In contrast, in Africa, countries such as Nigeria, Ethiopia, Ghana, Kenya, and Uganda are major producers. Nigeria leads with an estimated annual production of 1.2 million metric tons of moringa leaves. Ethiopia follows with 600,000 metric tons, while Ghana, Kenya, and Uganda contribute 200,000, 150,000, and 100,000 metric tons, respectively [6]. The production of moringa can be improved to meet the rising population growth, which is usually achieved by increasing plant density.

Encouraging farmers in suitable regions to cultivate moringa is essential for capitalizing on its benefits. Increasing plant density per hectare may help in addressing the growing pressure of increasing crop production on arable land in the face of a rising population and climate change [7,8]. A study by Mabapa et al. [9] examined the relationship between *M. oleifera* plant density and above-ground biomass production. Mabapa et al. [9] established an experimental site in Limpopo Province, South Africa: Syferkuil and Ofcolaco farms. Mabapa et al. [9] tested four plant densities: approximately 435,000, 300,000, 200,000, and 100,000 plants per hectare. The results demonstrated that the highest planting density (approximately 435,000 plants/ha) produced the greatest above-ground biomass. This effect was consistent across both study sites, with biomass yields ranging from 527 to 2867 kg/ha. Based on these findings, Mabapa et al. [9] recommend a planting density of approximately 435,000 plants per hectare to maximize moringa productivity. This suggests that, within the tested range, moringa may benefit from increased competition for resources, leading to more significant biomass accumulation [10]. Increasing plant density, while potentially boosting overall yield, can lead to negative impacts like reduced individual plant growth, decreased resource utilization, and increased competition for resources, ultimately impacting crop productivity and sustainability [11]. Another factor that limits the production of this medicinal plant is abiotic stress factors, such as salinity. Projections indicate that by the year 2050, more than half of the world’s arable land could be impacted by salinity, underscoring the urgency of addressing this significant agricultural challenge. The phenomenon of global warming has resulted in temperature and rainfall changes, as well as an expected increase in CO_2_ levels. These changes have caused a rise in saline stress, which has had detrimental impacts on agriculture worldwide [12].

Salinity, the buildup of soluble salts in soil or water, negatively affects crop growth and development when it reaches critical levels. This process can be categorized as either primary or secondary [12]. Soil saline–alkaline stress is a prominent environmental issue that contributes to substantial reductions in agricultural productivity, terrestrial biodiversity, and ecological well-being. Moringa exhibits restricted expression of its inherent genetic capacity for growth, development, and yield when exposed to elevated levels of salt in the soil. Consequently, this leads to a decrease in their commercial and economic worth. Primary salinization occurs naturally through processes like weathering of parent materials, sandstone deposition, and the influx of ocean water into coastal areas and rivers, leaving behind concentrated salts after evaporation [12]. Secondary salinization, on the other hand, stems from anthropogenic activities [12]. Deforestation, for example, disrupts soil structure and can lead to salt accumulation. Additionally, activities include using brackish or saline water for irrigation, poor land, and water management practices that lead to rising water tables. Seawater intrusion into coastal aquifers due to rising sea levels or over-extraction of freshwater. Excessive fertilizer application and inadequate drainage are factors that contribute to soil salinization in agriculture [12,13]. These factors can exacerbate salt buildup in the root zone, rendering the soil unproductive [13,14]. Waterlogging further compounds the problem by preventing the leaching of salts [14].

Among the factors that come with limited available arable land across the world, salinity challenges are a major hurdle that continue to hamper crop production as a result of climate variability. Soil salinity introduces significant challenges, such as ion toxicity, osmotic stress, and nutrient deficiencies, leading to oxidative stress and reduced plant productivity [15]. These stress conditions, exacerbated by high planting density, increase the production of reactive oxygen and nitrogen species, potentially leading to plant cell death [16]. A diverse array of microorganisms naturally colonizes plants, affecting their morphology, physiology, and biochemistry—and moringa is no exception [17]. One promising solution is the use of Arbuscular Mycorrhizal Fungi (AMF), known to alleviate both biotic and abiotic stress in most plants. These fungi form symbiotic relationships with over 80% of vascular plants, including moringa, and improve stress tolerance by enhancing nutrient absorption and reducing oxidative damage [18,19]. Arbuscular Mycorrhizal Fungi have been shown to enhance plant growth and salinity tolerance through various mechanisms, including regulating the host plant’s physiological and biochemical properties [20]. Extensive studies have demonstrated that AM fungi inoculation induces favorable physiological and biochemical modifications in plants, ultimately improving growth and yield under salinity stress [20]. This highlights the potential of AM fungi as an effective bio-ameliorative strategy for saline soils, enhancing the yield and quality of various economically significant plant species [21]. The fungi occupy two distinct environments: the root cortex and the surrounding soil, creating a physical connection to these two realms [22]. Within roots, they establish close interactions with the cortical cells, forming arbuscules or coils in the apoplastic space inside host cell walls [22]. Outside the roots, the fungi extend to over 15 cm away from the roots (depending on the species), interacting with mineral soil surfaces, soil organic matter, roots of other plants (potentially forming common mycorrhizal networks linking unrelated plants), and other various soil organisms [23]. In addition, the development of root architecture and nutrition, ion homeostasis, osmoregulation, antioxidant defense systems, and endogenous hormone regulation are the fundamental mechanisms behind AMF-enhanced salt tolerance of plants [23]. Despite the recognized importance of AMF in sustainable agriculture, their role in mitigating the combined effects of salinity and plant density stress on *M. oleifera* remains underexplored. This review aims to examine the literature on the use of AMF in alleviating these stress factors; AMF offer a promising direction for future research and the development of strategies to improve moringa cultivation in the face of growing environmental pressures.

## 2. The Crucial Role of Arbuscular Mycorrhizal Fungi in Sustainable Agriculture

**Figure 1:**
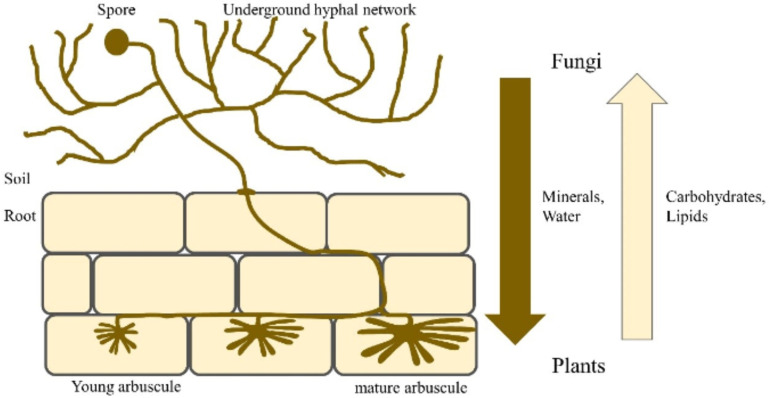
A simplified illustration depicting the colonization of a host plant’s roots by Arbuscular Mycorrhizal Fungi and the subsequent reciprocal exchange of resources between the symbiotic partners [40].

Mycorrhizal symbiosis, a ubiquitous phenomenon in terrestrial ecosystems, involves the formation of intricate associations between plant roots and specific fungal taxa [19]. Among the six recognized types of mycorrhizal associations [24,25], arbuscular mycorrhizae, established by fungi in the phylum *Glomeromycota*, stand out as the most prevalent, characterizing approximately 80% of aquatic and terrestrial plant species, including *Moringa oleifera* [26,27]. Some examples of these fungi include: *Funneliformis mosseae*, *Rhizoglomus intraradices*, *Rhizophagus fasciculatus*, *Acaulospora laevis*, *Acaulospora scrobiculata*, *Gigaspora margarita*, *Gigaspora rosea*, *Scutellaspora calospora*, and *Dentiscutata heterogama* [26,27]. It is commonly found in savannas or grasslands, subtropical and tropical forests, and agricultural fields [27,28]. This symbiotic relationship, characterized by the presence of fungal arbuscules within plant root cortical cells, facilitates a mutually beneficial exchange of resources [29]. Arbuscular Mycorrhizal Fungi (AMF) in the soil produce spores that germinate and infect the root systems of host plants [24,29]. These fungi form arbuscules within the plant’s root cortical cells after infection. As shown in Figure 1, arbuscules are specialized structures that facilitate nutrient exchange between the plant and the fungi, such as carbon, nitrogen, phosphate, potassium, and sulfate. Additionally, symbiosis is characterized by the formation of an extensive mycorrhizal network that extends from the plant roots into the surrounding soil [30].

Arbuscular Mycorrhizal Fungi (AMF), obligate biotrophs, derive essential carbon compounds, primarily carbohydrates and sugars, from their host plants [31]. In return, AMF provide a multitude of ecological services to their host species [32]. These benefits stem from the unique architecture and function of the mycorrhizal interface, which serves as a conduit for the bidirectional transfer of nutrients, signaling molecules, and protective compounds [33]. One of the well-documented benefits of AMF colonization is the enhanced uptake of soil nutrients, particularly phosphorus, which is often a limiting factor for plant growth [34]. This improved nutrient acquisition efficiency can be attributed to the extensive hyphal network of AMF, which extends far beyond the root zone, accessing nutrient-rich soil microsites inaccessible to plant roots [34,35]. In return for this enhanced nutrient supply, plants allocate up to 30% of their photosynthetically fixed carbon to their AMF partners [36]. Additionally, recent research highlighted the importance of lipids as a significant carbon source provided by plants to support AMF colonization and growth [37]. Beyond nutrient acquisition, AMF confer increased tolerance and resilience to various abiotic and biotic stressors, including drought, salinity, heavy metal toxicity, and intraspecific competition [38,39]. This heightened stress tolerance is attributed to a combination of factors, including improved water relations, osmotic adjustment, and antioxidant enzyme activity. The overall soil microbial diversity, including fungi and bacteria, can affect performance in moringa. *Pseudomonas* and *Bacillus* species sustain AMF through growth promotion and enhanced colonization [38].

Mycorrhizal fungi serve as intermediaries, shaping the interactions between plants and the diverse microorganisms inhabiting the soil [19]. This includes mediating relationships with both beneficial microbes, such as nitrogen-fixing bacteria and phosphorus-solubilizing fungi, and harmful pathogens [41,42]. By influencing the composition and activity of the rhizosphere microbiome, mycorrhizal fungi indirectly shape plant traits related to nutrient and water uptake, growth, and stress tolerance [43]. Extensive Common Mycorrhizal Networks (CMNs) physically connect plants within and between species, facilitating the transfer of nutrients and signaling molecules [44,45]. This interconnectedness allows for resource sharing, competition, and even communication between plants, ultimately influencing community dynamics and ecosystem processes [46]. Therefore, mycorrhizal fungi, along with other biotic and abiotic factors, act as key drivers of below-ground ecosystems, shaping plant communities and influencing the overall functioning of terrestrial ecosystems [43]. A study on *Moringa oleifera* with AMF demonstrated an increase in copper (Cu) and zinc (Zn) levels in the leaves, while other nutrients such as manganese (Mn), iron (Fe), and molybdenum (Mo) remained unchanged [47]. Inoculation of *Moringa oleifera* with Arbuscular Mycorrhizal Fungi (AMF) has been shown to significantly enhance plant growth, biomass production, and overall vigor. Studies report notable improvements in stem diameter, plant height, and biomass accumulation in AMF-inoculated moringa, demonstrating the positive influence of AMF on plant performance. A study conducted by Rubio-Sanz and Jaizme-Vega [48], in particular, observed a 23% increment in stem diameter and a 63% increment in shoot biomass accumulation for AMF-inoculated moringa as compared to non-mycorrhized moringa. Moreover, their results showed that mycorrhization with *F. mosseae* lead to an increase in the concentration of six macronutrients (N, P, K, Ca, Mg, and Na) and three micronutrients (Fe, Zn, and Mn) within *M. oleifera* leaves, revealing a four-fold increment in iron (Fe), two-fold increment in phosphorus (P) and potassium (K), and a 55% increment in nitrogen (N) [48]. The P tends to increase as AMF form a close symbiotic relationship with plants through the fungal hyphal networks. The ability of AMF to increase Fe depends on the AMF species. This beneficial symbiosis enhances nutrient bioavailability and uptake efficiency, as AMF improve the assimilation of essential nutrients and water, supporting rapid growth and robust plant development [48]. Furthermore, the improved nutrient status conferred by AMF inoculation strengthens moringa’s physiological resilience to environmental stressors, such as salinity and high planting densities, which are common challenges in sustainable agriculture. By enhancing moringa’s tolerance to salinity stress and density-induced competition, AMF not only optimize growth but also promote ecological and sustainable cultivation practices. The early-stage association of AMF with fast-growing trees like *Moringa oleifera* serves as an effective strategy for producing nutrient-rich, high-quality food products for human consumption [48]. Additionally, managing crop fertilization and employing techniques to enhance ecological and agroecological practices contribute to the advancement of sustainable agriculture while ensuring the production of superior-quality agricultural supplies [48]. The role of AMF in sustainable agriculture is paramount due to its numerous benefits. Proper management and maintenance of AMF populations in agricultural soils can significantly increase crop productivity, reduce fertilizer inputs, and boost environmental sustainability [49].

## 3. *Moringa oleifera* as a Multifaceted Plant

*Moringa oleifera*, a fast-growing deciduous tree species belonging to the family Moringaceae, is characterized by its robust growth habit, distinctive thick, tuberous roots, vibrant light green foliage, and prolific flowering culminating in elongated, pendulous fruits containing numerous seeds [50]. While indigenous to the sub-Himalayan regions of India, *M. oleifera* has been introduced and naturalized across diverse tropical and subtropical regions, including Southwest Asia, Northwest and Southwest Africa, Madagascar, the Caribbean Islands, the Philippines, and parts of the Americas, Asia, and the Middle East [51,52]. This widespread distribution is a testament to the species’ adaptability and multifaceted applications in agriculture, medicine, and industry [50].

Historically, *M. oleifera* has been an integral component of traditional horticulture, particularly in cities along the Pacific coast of Mexico, where it was primarily cultivated for ornamental purposes [53]. However, the recognition of its nutritional and medicinal properties, attributed to the rich composition of proteins, vitamins, minerals, and carotenoids found in all parts of the plant, has led to a surge in its cultivation and utilization globally, particularly since the 1990s [53]. Despite its resilience, *M. oleifera*, like many crops, faces significant challenges in agricultural settings due to the increasing prevalence of abiotic and biotic stressors, often occurring simultaneously [54]. Salinity and high planting densities, for instance, are common stressors that can significantly impact *M. oleifera* growth and productivity [55]. In this context, the synergistic benefits of Arbuscular Mycorrhizal Fungi (AMF) emerge as a promising avenue for sustainable moringa cultivation. Arbuscular Mycorrhizal Fungi (AMF) not only improve nutrient uptake, particularly phosphorus, but also bolster plant tolerance to abiotic stresses like salinity and biotic stresses like high plant density [38]. Notably, AMF form intricate interactions with other beneficial soil microorganisms, including nitrogen-fixing bacteria, creating a symbiotic network that further amplifies plant resilience [56]. Further research is warranted to fully elucidate the intricate interplay between AMF and M. oleifera under combined stress conditions, paving the way for harnessing the full potential of this symbiotic relationship to increase moringa productivity and resilience in a changing world [57].

## 4. Moringa Ethnomedicinal Use and Pharmacological Activities

*Moringa oleifera* has a rich history of use in traditional medicine, with various parts of the plant employed to address a wide array of ailments, as illustrated in Table 1. The leaves, for instance, have been traditionally used to manage conditions such as diabetes, hypertension, genito-urinary disorders, arthritis, typhoid fever, parasitic infections, and skin diseases [53]. On the other hand, flowers have been incorporated into traditional remedies for spleen enlargement, tumors, hysteria, muscular disorders, and inflammations, and are also believed to possess aphrodisiac properties.

Beyond traditional practices, scientific investigations—both in vitro and in vivo (animal models)—have provided evidence supporting the potential therapeutic benefits of *M. oleifera* leaf, seed, and root extracts [64]. These studies suggest potential anticancer, hepatoprotective, hypoglycemic, anti-inflammatory, antibacterial, antifungal, antiviral, and anti-sickling properties associated with *M. oleifera*. Furthermore, research highlights potential benefits in managing Alzheimer’s disease, stomach ulcers, high cholesterol, and promoting wound healing, as documented by the Memorial Sloan Kettering Cancer Center [53]. *Moringa oleifera* stands out not only for its diverse medicinal applications but also for its remarkable nutritional value and industrial potential [65,66]. Research has highlighted its rich composition, containing significant levels of vitamin C, vitamin A, and essential amino acids, underscoring its importance as a valuable dietary supplement [67]. This nutritional richness is further exemplified by comparisons with common foods: *M. oleifera* leaves possess twice the protein content of yogurt, four times the vitamin A content of carrots, three times the potassium content of bananas, seven times the vitamin C content of oranges, and four times the calcium content of milk [68]. Beyond its nutritional significance, *M. oleifera* exhibits remarkable resilience, particularly its high drought tolerance compared to other plant species. This characteristic makes it an invaluable resource in arid and semi-arid regions, especially during dry seasons when other crops struggle to thrive [67]. Furthermore, *M. oleifera* has demonstrated potential in agricultural applications. The high concentration of zeatin, a natural plant growth regulator found in M. oleifera, has been explored for its ability to increase crop yields [69]. The applications of M. oleifera extend beyond nutrition and agriculture. Its antimicrobial properties have been utilized in water purification processes, where it acts as a natural coagulant [70]. Moreover, the high oleic acid content in *M. oleifera* seed oil makes it suitable for a variety of industrial uses, including edible oil, cosmetics, biodiesel production, and even as a lubricant for machinery [71].

*Moringa oleifera* is a rich source of essential nutrients and bioactive compounds. Its various parts, including leaves, seeds, roots, flowers, gum, bark, and fruit pods, contain an abundance of vitamins, minerals, amino acids, β-carotene, omega-3 and omega-6 fatty acids, and antioxidants [72]. Phytochemical analysis of *M. oleifera* has revealed diverse compounds, broadly categorized as flavonoids, carbamates, glucosinolates, phenols, steroids, and carotenoids [53,73]. Flavonoids, primarily present as glucosides, are predominantly found in the leaves, with quercetin and kaempferol being the most abundant representatives. The identified flavonoids belong to the classes of flavonols and isoflavones [73]. Carbamates, another class of bioactive compounds, are also present in *M. oleifera*. Phenolic compounds, including their esters and glycosides, have been isolated from both the leaves and seeds. β-Carotenoids, essential micronutrients known for their role in disease prevention and immune support, are also found in *M. oleifera* [73]. Furthermore, the leaves are a notable source of omega-3 and omega-6 polyunsaturated fatty acids [72]. The accumulation of bioactive compounds within *M. oleifera* is prone to variation depending on environmental factors such as light availability, rhizosphere, competition for resources, and soil salinization [73]. Abiotic stresses, such as salinity stress, serve as potent elicitors of secondary metabolite production in plants by directing energy toward defense mechanisms through the activation of specific biosynthetic pathways [74]. Although it possesses deleterious effects, salinity remains one of the major factors influencing plant physiology, biochemistry, and the synthesis of bioactive compounds in medicinal plants. Contrariwise, several studies have shown that moderate salinity stress in medicinal plants can enhance the synthesis of bioactive compounds, such as polyamines, which assist in scavenging reactive oxygen species (ROS) [75]. Bistgani et al., [76] demonstrated that treatment with 60 mM NaCl resulted in approximately a 20% increase in total phenolic content in *Thymus vulgaris* (L.) and *T. daenensis* Celak, while leaf flavonoid content rose by 38.6% and 36.6% in response to 60 and 90 mM NaCl, respectively. Similarly, Boughalleb et al. [77] reported a marked enhancement in total flavonoid compounds, phenolic acids, and phenolic compounds in *Polygonum equisetiforme* under a salinity level of 300 mM NaCl [78]. Salinity stress was also shown to improve antioxidant potential in *M. oleifera* by elevating compounds such as ascorbic acid, glutathione, total phenols, and flavonoids, thereby enhancing their biological activities as per the study conducted by Azeem et al. [78]. A significant linear increase in glutathione levels was observed at (50 mM NaCl), moderate salinity (40%), and even more prominently at (100 mM NaCl), indicating a high salinity of 127%. Conversely, ascorbic acid was elevated primarily under high salinity conditions at 13%. The study reported how total phenols remained stable at moderate salinity (50 mM NaCl) but increased significantly under high salinity (100 mM NaCl) by 26%. Similarly, total flavonoids demonstrated a steady increase (21–60%) as salinity levels rose, underscoring their importance in mitigating oxidative stress. Leaf pigments like betacyanins, indicaxanthin, and carotenoids shield cells from salt-induced photo-damage by reducing the pressure on chlorophyll, which otherwise lowers the plant’s ability to capture light [78]. The study by Azeem et al. [78] observed that under moderate salinity stress of 50 mM NaCl, moringa exhibited a significant increase in betacyanins, while indicaxanthin showed a slight increase, and carotenoids remained unchanged, suggesting that plants managed photo-damage and oxidative stress more efficiently. However, when salinity stress becomes excessive, it can have detrimental effects on *M. oleifera*, for example, by reducing chlorophyll and carotenoids [78]. The study by Azeem et al. [78] supports these claims as under high salinity stress of 100 mM NaCl, moringa experienced a decrease in carotenoid levels, indicating a diminished ability to absorb and transfer light energy and to dissipate heat. In such cases, betacyanin and indicaxanthin help reduce salt-induced oxidative damage, which explains their increased levels. Moreover, under high salinity stress, toxic ions sodium (Na^+^) and chloride (Cl^−^) build up in various parts of the plant, which reduces its growth and productivity. Elhag and Abdalla [79] observed that as salinity increases, sodium levels go up and potassium (K^+^) levels drop in different plant tissues. While this ion buildup can help the plant draw in water by enhancing the osmotic gradient, excessive Na^+^ and Cl^−^ taken up by the roots limits the availability of _K^+^ and other essential minerals such as calcium (Ca^2+^), magnesium (Mg^2+^), nitrogen (N), and phosphorus (P). Additionally, the above-ground parts of the plant may experience K^+^ deficiency because less potassium is transported from the roots under high salinity conditions [78]. Since potassium is crucial for plant growth during stress, reduced uptake due to high sodium levels can cause ion competition on potassium transport sites [80]. This scenario can induce membrane depolarization and compromise plasma membrane integrity, thereby displacing essential minerals (such as K^+^, Ca^2+^, Mg^2+^, etc.) and water [81]. Salinity significantly disrupts the uptake of essential nutrients, resulting in imbalanced mineral content within the plant [82]. High salt concentrations inhibit the absorption of vital elements such as iron (Fe), calcium (Ca), potassium (K), zinc (Zn), boron (B), and magnesium (Mg) [82]. In the leaves, this stress leads to reduced levels of nitrogen (N), K, and Zn, while in the roots, the uptake of phosphorus (P), K, Ca, and Mg is notably diminished [83]. Moreover, salinity alters the soil’s osmotic potential, making it more difficult for plant roots to absorb mineral nutrients effectively [84]. The increased ion toxicity under high salinity further exacerbates nutrient imbalances by reducing the uptake of both macronutrients, such as P, K, and Mg, and micronutrients, including Fe, Zn, copper (Cu), and manganese (Mn). Consequently, these disruptions lead to mineral deficiencies and adversely affect the interactions between the soil and the plant microbiome [85].

### 4.1. Flavonoids

**Figure 2:**
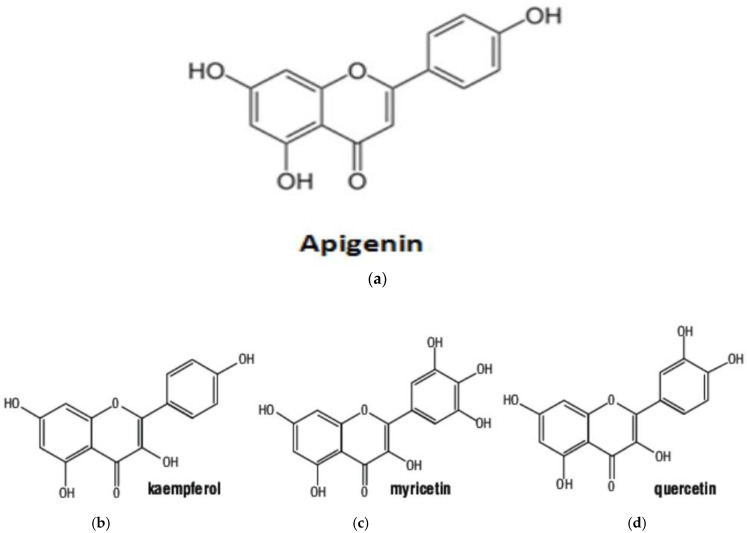
Structural formulae of flavonoid matrices present in *M. oleifera*: (**a**) apigenin; (**b**) kaempferol; (**c**) myricetin (**d**) and quercetin [87].

The moringa genus exhibits high antioxidant activity, primarily attributed to its rich flavonoid content. Flavonoids in this genus predominantly occur as flavanols and glycosides [66]. Notable examples include rutin, quercetin, rhamnetin, kaempferol, apigenin, and myricetin Figure 2. Studies have focused on optimizing flavonoid extraction from *M. oleifera*, with subcritical ethanol extraction demonstrating superior yields compared to traditional reflux methods [85,86].

### 4.2. Phenolic Acids

**Figure 3:**
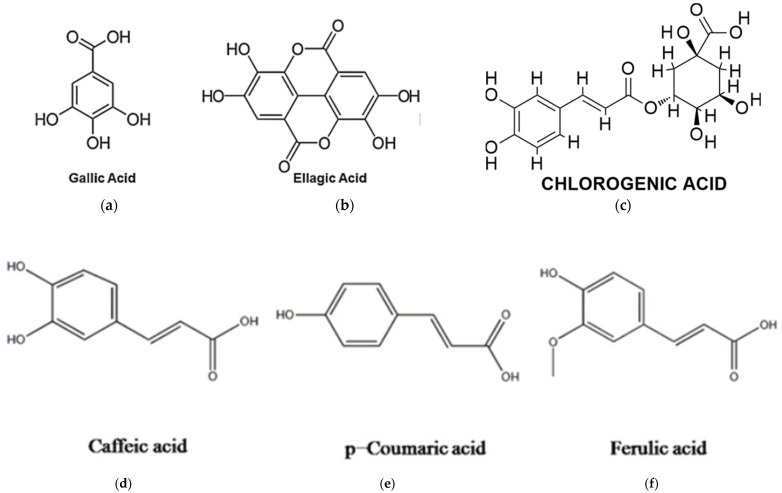
Structural formulae of phenolic acids matrices present in *M. oleifera*: (**a**) gallic acid; (**b**) ellagic acid; (**c**) chlorogenic acid; (**d**) caffeic acid; (**e**) p-coumaric acid; (**f**) ferulic acid [78].

*Moringa oleifera* leaves are particularly rich in phenolic acids, with gallic acid being the most abundant. Other phenolic acids detected in the leaves include ellagic acid, ferulic acid, caffeic acid, o-coumaric acid, and chlorogenic acid, as indicated in Figure 3. Gentisic acid, syringic acid, ρ-coumaric acid, and sinapic acid have also been identified in trace amounts [69,88].

### 4.3. Glucosinolates

**Figure 4:**
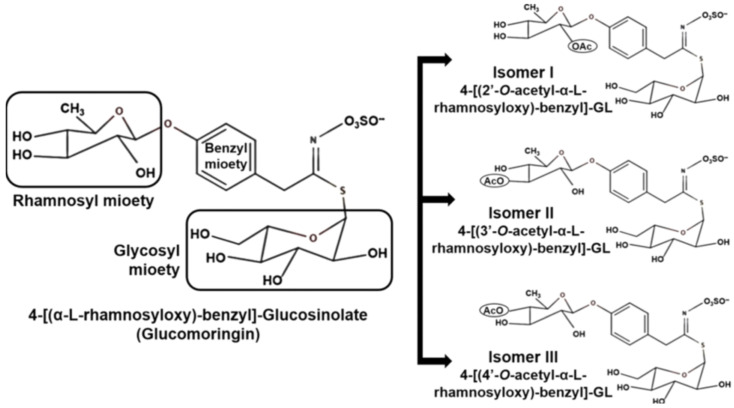
Characteristic glucosinolates of *Moringa oleifera* tree: 4-[(α-L-rhamnosyloxy)-benzyl]-glucosinolate, commonly known as glucomoringin, and its three isomers. Circles in the isomers indicate acetylation [94].

*Moringa* species are characterized by an abundance of glucosinolates. The predominant glucosinolate is 4-O-(αL-rhamnopyranosyloxy)-benzyl glucosinolate, commonly known as glucomoringin as depicted in Figure 4. Three isomers of 4-O-(α-L-acetylrhamnopyrosyloxy)-benzyl glucosinolate have also been detected in *M. oleifera* leaves, with their presence influenced by leaf maturity and physiological state [89,90]. Tissue disruption (such as cutting or chewing) activates myrosinase, an enzyme that catalyzes the conversion of glucosinolates to isothiocyanates. The most abundant isothiocyanate in moringa is 4-[(α-L-rhamnosyloxy) benzyl] isothiocyanate, derived from glucomoringin. Isothiocyanates have garnered significant research interest due to their diverse biological activities, including anticancer, antidiabetic, antimicrobial, and anti-inflammatory effects [91,92,93].

### 4.4. Terpenes

**Figure 5:**
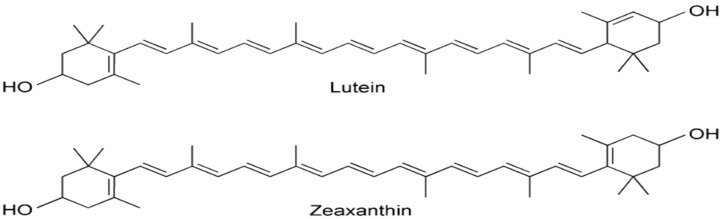
Chemical structures of terpenes present in *Moringa oleifera*: lutein and zeaxanthin [97].

Lutein is the major carotenoid found in *M. oleifera* leaves [95,96]. Interestingly, α-carotene, commonly present in green leafy plants, is not detected in *M. oleifera*, suggesting its complete conversion to lutein as indicated by Saini et al. [95]. Other carotenoids identified include all E-luteoxanthin, 13-Z-lutein, 15-Z-β-carotene, and all-E-zeaxanthin as demonstrated in Figure 5 [96].

### 4.5. Alkaloids

**Figure 6:**
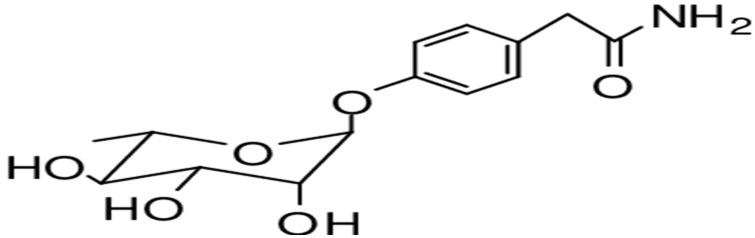
Chemical structure of marumoside A [100].

*Moringa oleifera* contains various alkaloids, including marumoside A, marumoside B, and pyrrolemarumine-4-*O*-α-l-rhamnopyranoside. Alkaloids, particularly those with N,α-l-rhamnopyranosyl structures, have demonstrated cardioprotective effects against hypertension. Furthermore, analysis of MO’s bark and leaves revealed two primary sterols: β-sitosterol-2-*O*- β-d-galactopyranoside and β-sitosterol, as shown in Figure 6 [98,99]. These sterols exhibit anti-inflammatory properties by suppressing the release of inflammatory factors.

### 4.6. Sterols

**Figure 7:**
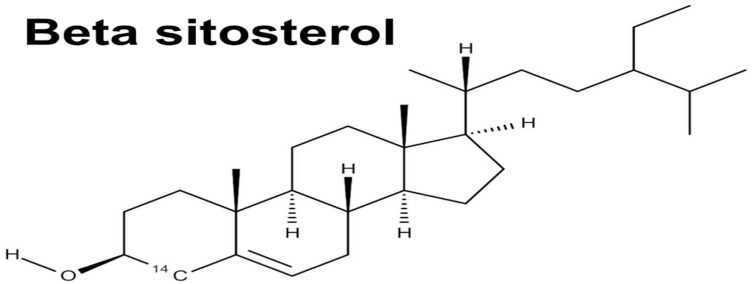
Structural formulae of β-Sitosterol [94].

β-Sitosterol-3-O-β-D-galactopyranoside, a sterol glycoside, has been isolated from the stem bark of *M. oleifera*, as shown in Figure 7 [101]. Moreover, the isolation of β-Sitosterol in *M. oleifera* was successful on the leaves and seeds of *Moringa oleifera*, showcasing the diversity of phytochemical composition within different parts of the plant.

## 5. *Moringa oleifera*’s Adaptive Strategies Against Salinity

Salinity stress poses a significant threat to global agriculture, compromising crop productivity and jeopardizing food security [102,103]. Understanding and mitigating the detrimental effects of salinity is crucial for developing sustainable agricultural practices to meet current and future food demands. Moringa can mitigate mild salinity (50 mM NaCl) through the preservation of succulence, weight ratios, and biomass distribution patterns in both the shoot and root [102,103]. The phenological features in moringa exhibit a significant decrease under conditions of high salinity (100 mM NaCl). Salinity negatively impacts both the morphology and physiology of moringa. It hinders seed germination, stunts growth and development, and ultimately reduces yield [104]. To counter these negative impacts, the integration of AMF is essential, as it enhances nutrient and water uptake, strengthens the rhizosphere, and mitigates oxidative stress [22]. Soil, being composed of mineral constituents such as sand, silt, and clay [105], has properties that directly influence its porosity and hydraulic characteristics. Arbuscular Mycorrhizal Fungi (AMF) play a crucial role in mitigating the negative consequences of salinity on soil microbiomes and the microbial communities associated with *M. oleifera*. Salinity often disrupts soil structure, reduces water availability, and impairs microbial activity, which collectively challenges plant growth and soil health [106]. However, AMF, due to their unique abilities, can alleviate these effects by enhancing soil structure and fostering microbial connectivity [106].

Soil, being composed of mineral constituents such as sand, silt, and clay [105], has properties that directly influence its porosity and hydraulic characteristics, which, in turn, shape the soil microbiome [107]. AMF effectively bridge soil pores and facilitate the formation of soil aggregates through their physical strength and the exudation of glomalin-related soil proteins [108]. These aggregates improve soil stability and resilience to salinity-induced structural degradation.

Moreover, AMF hyphae act as biological highways, forming extensive networks that enhance the connectivity of soil pores. This connectivity allows for capillary water movement along hyphal bridges, which is especially critical in saline soils where water availability is limited. These fungal highways also enable bacteria to traverse through the soil matrix to reach new microhabitats, promoting a dynamic and diverse microbial community even under saline conditions [109,110].

In the context of *Moringa oleifera*, AMF mitigate salinity stress by improving nutrient uptake, particularly phosphorus and nitrogen, and facilitating water absorption through enhanced soil–water interactions [111]. Additionally, the improved microbial mobility and resilience supported by AMF contribute to the stabilization and maintenance of a functional soil microbiome, ensuring the sustainability of plant–microbe interactions even in challenging saline environments [38]. Consequently, AMF serve as a cornerstone in managing salinity-related challenges, promoting both soil and plant health. Soil pH is widely recognized as a key factor influencing soil microbial communities, with significant implications for both bacterial transport and fungal interactions [38]. In the context of saline soils, the negative consequences of salinity, such as soil acidification or alkalization, can have far-reaching effects on soil pH, microbial dynamics, and plant health [38]. For Arbuscular Mycorrhizal Fungi (AMF), these pH alterations present unique challenges and opportunities for mitigating the detrimental effects of salinity.

Arbuscular Mycorrhizal Fungi (AMF) play a critical role in modulating the pH of their hyphosphere, a trait that is particularly significant in saline environments where pH shifts can limit nutrient availability and microbial activity [38]. Arbuscular mycorrhizal fungal hyphae can influence the pH of their surrounding soil by altering phosphorus availability and phosphatase activity, which may create localized conditions more favorable for bacterial colonization and activity [87,112]. By regulating these micro-environmental conditions, AMF may mitigate the impacts of salinity-induced pH extremes, promoting more stable microbial interactions within the soil.

Environmental factors such as pH also directly affect AMF development, with differential tolerance observed among fungal families [113,114]. For instance, species like *Glomus* sp. and *Rhizophagus intraradices* show reduced hyphal growth under acidic pH conditions [112,115]. This reduction in hyphal growth may limit the capacity of AMF to act as “highways” for bacterial transport, potentially curtailing the protective and nutrient-translocating roles of AMF in saline soils.

The alteration of soil pH caused by salinity can also negatively impact plants such as *Moringa oleifera*, a species known for its high nutritional and economic value. Changes in pH can hinder the availability of essential nutrients like phosphorus, iron, and zinc, which are critical for moringa growth and productivity [116]. Furthermore, acidic pH conditions may restrict the movement of beneficial bacteria along fungal hyphae, as observed in other mycorrhizal systems [117]. Conversely, alkaline conditions may compromise nutrient solubility and uptake, further exacerbating stress in moringa [116].

Arbuscular Mycorrhizal Fungi (AMF) can serve as an important ally in combating these challenges. By stabilizing soil pH, enhancing nutrient availability, and fostering beneficial microbial interactions, AMF may help alleviate the negative impacts of salinity on soil and plant health [106]. However, the efficacy of these fungi depends on their ability to adapt to the specific pH conditions of the soil [106]. Therefore, selecting AMF species or strains with high tolerance to the prevailing soil pH, such as those naturally adapted to acidic or saline environments, may be crucial for optimizing their benefits to moringa cultivation under saline stress.

The photosynthetic machinery is particularly vulnerable to salinity stress [118]. Elevated salt concentrations disrupt chloroplast ultrastructure, impair the photosystem II complex, and reduce chlorophyll and carotenoid content, collectively leading to reduced photosynthetic efficiency [118]. Furthermore, salinity disrupts transpiration and gaseous exchange by decreasing stomatal conductance [118]. Moreover, salinity induces oxidative stress by increasing the production of free radicals, namely, reactive oxygen species, within plant cells. ROS inflict damage on cellular components, causing lipid peroxidation, membrane deterioration, and damage to DNA and proteins [118,119].

Osmotic stress is another major consequence of salinity. High salt concentrations in the soil lower both soil water potential and leaf water potential, disrupting plant water relations and reducing turgor pressure [120]. *Moringa* plants, like other crops, absorb salt from the soil through specialized transporters, leading to ion toxicity. The excessive accumulation of sodium (Na^+^) and chloride ions (Cl^−^) disrupts mineral uptake, particularly potassium (K^+^) and calcium (Ca^2+^), resulting in ionic imbalance [121].

El-Dabh et al. [122] found that subjecting moringa plants to salinity stress during their early growth stage, using a 1:1 NaCl and CaCl_2_ solution, diminished growth parameters. While relatively low salt concentrations caused a slight decrease in plant height and stem diameter, leaf development and branch count remained unaffected [122]. This contrasts with other medicinal plants like *Erthrina variegata*, which showed no growth alterations at the seedling stage under low salinity conditions [123]. In fact, low salt concentrations have been shown to stimulate growth in certain tree species, including *Eucalyptus camadulensis* and *Dalbergia sisso*, and promote stem and root elongation in *Cassia montana* [124,125].

Conversely, increased salinity negatively impacted *Moringa oleifera*, reducing plant height, stem diameter, branch and leaf count, and root length [126]. These findings align with previous studies on *Albezia lebbeck* and *Melia azederach*, where seedling growth was similarly hampered by elevated salinity [127]. This growth inhibition under high salinity is attributed to the reduced water and osmotic potential within the plants [128]. While salinity’s impact on plant water uptake and subsequent growth reduction has been widely acknowledged, recent research suggests that hormonal signals originating from the roots may play a role in inhibiting shoot growth [129].

The study by El-Dabh et al. [122] further revealed that high salinity negatively affected both shoot and root dry weight in *M. oleifera*. Similar observations have been made in *Prosopis cineraria* and *Cassia angustifolia* [130,131]. Additionally, chlorophyll and carotenoid levels were impacted by salinity, declining steadily as salt concentration increased [122]. This aligns with previous findings in other tree species, where high salinity led to reduced pigment content in *Erythrina variegate* [124], decreased chlorophyll concentration in *Eucalyptus camadulensis*, and similar effects in *M. oleifera* [122].

### 5.1. Salinity Stress Detrimentally Impacts Moringa Growth and Physiology

#### 5.1.1. Physiological and Biochemical Adaptations

- Ion Homeostasis and Compartmentalization: Moringa maintains cellular ion balance by regulating the uptake, transport, and sequestration of ions, particularly sodium and chloride. This minimizes the toxic effects of excessive ion accumulation in sensitive cellular compartments [132].
- Osmotic Adjustment: To counter the osmotic stress induced by high salt concentrations, moringa accumulates compatible solutes or osmolytes, such as proline, glycine betaine, and sugars. These osmolytes help maintain cell turgor and protect cellular components from damage [132].
- Antioxidant Defense System: Salinity stress triggers the overproduction of reactive oxygen species, which can damage cellular components. Moringa enhances its antioxidant defense machinery, including enzymes like superoxide dismutase, catalase, and peroxidase, to scavenge ROS and mitigate oxidative stress [132].
- Polyamine Biosynthesis: Polyamines, such as putrescine, spermidine, and spermine, play a crucial role in stress tolerance. Moringa increases polyamine biosynthesis under salinity stress, contributing to ROS scavenging, membrane stabilization, and ion homeostasis [132].
- Morpho-Anatomical Modifications: Moringa exhibits structural changes in response to salinity, including alterations in root architecture, leaf morphology, and the size and number of organelles like chloroplasts, mitochondria, and peroxisomes. These modifications improve water uptake, reduce transpiration, and improve overall stress tolerance [133].

#### 5.1.2. Molecular Adaptations

- Phytohormone Regulation: Salinity stress triggers changes in phytohormone levels in moringa. Hormones like abscisic acid, auxins, cytokinins, salicylic acid, jasmonic acid, gibberellins, and brassinosteroids play crucial roles in regulating plant responses to salinity, including stomatal closure, root development, and stress signaling [65].
- Gene Expression and Omics Approaches: Moringa activates a complex network of genes, transcription factors, and proteins to combat salinity stress [134]. Omics approaches, such as genomics, transcriptomics, proteomics, and metabolomics, have provided valuable insights into the molecular mechanisms underlying salinity tolerance in moringa, paving the way for developing salt-tolerant varieties and improving crop productivity [134].

Under salinity stress, *M. oleifera*, like other plants, experiences an imbalance in cellular redox homeostasis, leading to oxidative stress. This stress arises from the overproduction of reactive oxygen species, including singlet oxygen, superoxide ions, and hydrogen peroxide [135]. These highly reactive molecules damage essential cellular components such as proteins, lipids, nucleic acids, and cellular membranes, including those of vital organelles like mitochondria and chloroplasts [136]. The photorespiration process, while essential for plant metabolism, can exacerbate oxidative stress by generating hydrogen peroxide during the oxidation of glycolate [137]. To combat this oxidative damage, *M. oleifera* has evolved a sophisticated antioxidant defense system comprising enzymatic and non-enzymatic components.

#### 5.1.3. Enzymatic Antioxidants

- Superoxide Dismutase: Acting as the first line of defense, SOD catalyzes the rapid dismutation of superoxide radicals (O_2^−^_) into the less reactive hydrogen peroxide (H_2_O_2_) and molecular oxygen (O_2_) [138]. Though hydrogen peroxide is less reactive, it still threatens cellular integrity if not further detoxified [138]. The importance of SOD lies in its rapid neutralization of superoxide radicals, preventing them from interacting with essential cellular components like DNA, proteins, and lipids. This is particularly important because unchecked superoxide radicals can lead to oxidative damage, which could result in mutations, protein dysfunction, and lipid peroxidation [138].
- Catalase and Ascorbate Peroxidase: Hydrogen peroxide is produced as a byproduct of SOD’s action, and though it is less reactive than superoxide radicals, it can cause oxidative damage [139]. Catalase and ascorbate peroxidase (APX) are responsible for eliminating H_2_O_2_, preventing it from causing further harm [139]. Catalase is highly efficient in converting H_2_O_2_ into water (H_2_O) and oxygen (O_2_), which are harmless to cells. This reaction is critical in protecting peroxisomes, where high levels of hydrogen peroxide may accumulate due to fatty acid metabolism [139]. Ascorbate peroxidase (APX) functions in conjunction with the ascorbate–glutathione cycle to detoxify hydrogen peroxide. It uses ascorbate (vitamin C) as an electron donor to convert H_2_O_2_ to H_2_O, with dehydroascorbate as the byproduct. This enzyme is particularly important in chloroplasts and other cellular compartments with high oxidative stress [139].
- Glutathione Peroxidase: Glutathione peroxidase provides an additional layer of protection against oxidative damage by targeting hydrogen peroxide and lipid peroxides [140]. When ROS attack cell membranes, they cause lipid peroxidation, which damages the cell structure and function of the lipid bilayer [132]. GPX reduces these lipid peroxides to their corresponding alcohol and water, restoring membrane integrity and further cellular damage [140]. In addition to detoxifying lipid peroxides, GPX also reduces hydrogen peroxide in the presence of reduced glutathione (GSH), converting it into water [140]. This reaction protects cellular membranes and ensures that harmful ROS do not accumulate to toxic levels [140]. The Fenton reaction, which involves the interaction of hydrogen peroxide with transition metals like iron, can produce highly reactive hydroxyl radicals. GPX mitigates this by detoxifying hydrogen peroxide before it can participate in this reaction, thus minimizing hydroxyl radical formation and, consequently, the resulting oxidative stress [140].

#### 5.1.4. Non-Enzymatic Antioxidants

- Ascorbic Acid: This potent antioxidant acts as an electron donor, contributing to the ascorbate–glutathione cycle, which efficiently reduces H_2_O_2_ to H_2_O using APX. Ascorbic acid is also involved in zeaxanthin production during the xanthophyll cycle and tocopherol synthesis, which contribute to moringa stress tolerance [141].
- Glutathione: Glutathione (GSH) is a small, tripeptide molecule comprising glutamine, cysteine, and glycine. It serves multiple roles in maintaining the redox balance of cells and serves as a pivotal reduced glutathione (GSH) and oxidized glutathione (GSSG) [142]. The ratio of GSH to GSSG is critical for determining the redox state of the cell. In healthy, non-stressed cells, GSH predominates; this balance is essential for neutralizing ROS. When oxidative stress occurs, such as under salinity conditions, GSH reacts with ROS to form GSSG, effectively reducing the ROS levels and protecting cellular components from oxidative damage [142]. The ability of glutathione to maintain cellular redox homeostasis is vital for protecting proteins, lipids, and DNA from ROS-induced damage. Under conditions of high salinity, where ROS production tends to be elevated due to disrupted cellular metabolism and ionic imbalances, glutathione’s role in scavenging harmful species becomes even more critical [142]. Plays a vital role in maintaining cellular redox balance and participates in the ascorbate–glutathione cycle [143].
- Phenolic Compounds: *Moringa oleifera* is rich in secondary metabolites like polyphenols and flavonoids, which act as potent antioxidants. These compounds effectively scavenge free radicals, halting oxidative chain reactions and protecting cellular macromolecules and membranes from damage [144].

*Moringa oleifera*, despite its impressive natural defenses against salinity, experiences a breaking point when stress becomes too severe [143]. The same antioxidant properties that allow it to thrive in moderately saline environments become insufficient under persistent, high salinity [143,144]. While these antioxidants can scavenge excess reactive oxygen species produced due to salt stress, their capacity is finite [145]. When salinity levels are too high or exposure is prolonged, the antioxidant system becomes overwhelmed. This leads to a cascade of negative effects, including cellular damage, impaired metabolism, and ultimately, growth inhibition or even death [132]. Essentially, moringa’s resilience is dependent on a balance between the severity of the stress and its physiological capacity to counteract it. Once this balance is tipped, even its robust adaptive mechanisms are no longer sufficient to ensure survival. This highlights the crucial role of sustainable biofertilizers like AMF in salinity alleviation. Arbuscular Mycorrhizal Fungi (AMF) provide a multifaceted approach to enhancing moringa’s salt tolerance. They not only boost nutrient uptake, compensating for salt-induced deficiencies, but also enhance the plant’s own osmoregulation mechanisms, helping maintain cellular water balance [146]. By bolstering these physiological processes, AMF can effectively raise moringa’s tolerance threshold, allowing it to thrive even in conditions that would otherwise prove detrimental [147]. Therefore, integrating AMF into cultivation strategies for moringa, especially in saline-prone areas, is not merely beneficial, but essential for ensuring optimal growth and productivity [141].

## 6. The Dual Role of Reactive Oxygen Species in *Moringa oleifera* Under Salinity Stress

While possessing destructive properties, reactive oxygen species (ROS) play a complex and multifaceted role in plant responses to salinity stress [148,149]. Although excessive ROS levels cause irreversible cellular damage, tightly regulated ROS production acts as a critical signaling mechanism, activating stress response pathways and enhancing tolerance in *Moringa oleifera* [149]. The inherent challenge in deciphering ROS signaling lies in the diverse and interconvertible nature of these molecules, making it difficult to discern their specific roles in cytotoxic versus adaptive responses [150].

### 6.1. ROS-Induced Damage

When salinity stress surpasses the tolerance threshold of moringa, the delicate balance between ROS production and scavenging is disrupted [143]. Despite activating antioxidant defense mechanisms, the plant’s capacity to neutralize ROS becomes overwhelmed, leading to oxidative stress [151]. This excess ROS inflicts damage on various cellular components, including proteins, lipids, and DNA, ultimately compromising cell integrity and leading to cell death [152].

### 6.2. ROS as Signaling Molecules

Paradoxically, controlled ROS production acts as a critical signaling cue, triggering adaptive responses to salinity stress [150]. These signaling events lead to morphological and physiological changes that increase stress tolerance [153,154]. The transient nature of ROS signaling is crucial; responses are rapidly activated upon stress perception and subside upon stress alleviation [155]. The interplay between ROS-induced damage and ROS-mediated signaling highlights the complexity of plant stress responses. Further research is crucial to unravel the intricate mechanisms governing ROS homeostasis and signaling specificity in *M. oleifera* to develop strategies for enhancing its salinity tolerance.

## 7. Plant Population Density and Resource Utilization

Planting density entails not only the number of individuals (plants) per unit area, but also the proximity of one plant to another [156]. The selection of plant density has a significant effect on yield per unit area, light interception, and resource use efficiency [157]. This is attributed to the intraspecific competition for above-ground resources (space and light) and below-ground resources (water and nutrients) in the rhizosphere [157]. Planting density influences the light interception of plants and, thus, their photosynthetic activity and overall biomass production. There is a positive correlation between the interception of photosynthetically active radiation (PAR) and biomass production [152,158,159]. The less light available, the lower the plant growth potential. Arbuscular Mycorrhizal Fungi form vast networks of hyphae that extend well beyond the plant’s own roots, effectively expanding the available surface area for absorption [160]. This extensive network acts as an extension of the root system, accessing water and essential nutrients from soil regions that roots alone might not reach [160]. As a result, plants are able to maintain a more consistent and efficient uptake of resources, which supports critical metabolic functions. Even when light availability is limited, a condition that typically hinders photosynthesis and reduces growth, the enhanced nutrient and water absorption provided by these hyphal networks can compensate for the deficit [160]. This support helps to sustain robust metabolic activities, ensuring that the plant continues to grow vigorously [160]. Ultimately, this leads to improved overall biomass production, even under suboptimal above-ground conditions [160]. Another key thing to remember is that by establishing a mutually beneficial relationship with plant roots, AMF enable a more efficient distribution and utilization of vital resources within the plant [160]. This enhanced resource allocation not only improves the uptake of nutrients and water but also triggers beneficial physiological responses that fortify the plant’s overall health [38]. As a result, moringa becomes more resilient and better equipped to handle the stress associated with reduced light availability in densely planted environments, ultimately supporting sustained growth and productivity.

High planting density environments can lead to variations in the quantity and quality of light, which can trigger changes in the plant’s photomorphogenesis. The shading effect caused by high planting densities changes light quality, resulting in increased far-red light and triggering hormonal changes that can result in the elongation of the stem cells of the plants being shaded [158]. This phenomenon is referred to as etiolation. Plant density plays an important role in regulating population growth, biomass partitioning, and nutrient acquisition [159]. The morphological changes that result from etiolation, such as excessive elongation of stem cells, can compromise plant structure and function. However, the enhanced nutrient and water absorption facilitated by AMF supports the plant’s metabolism and overall growth [53]. This improved nutritional status helps maintain a more balanced growth pattern, reducing the abnormal elongation that typically occurs under shaded conditions, and thereby promoting sturdier and healthier *M. oleifera* plants [161]. Higher plant densities can lead to greater competition for resources among individuals within a population. This, in turn, can modify plant structures and physiological traits by affecting the quantity of resources, such as light, water, and nutrients, available to each individual [162,163]. In densely planted moringa, the close proximity of plants and reduced airflow create favorable conditions for pest infestations [160]. This scenario not only increases the likelihood of pests, such as aphids, but also amplifies the stress on plants by disrupting their normal physiological processes [160]. However, AMF offer a promising solution to reverse these adverse effects through several interlinked mechanisms.

AMF have been shown to strengthen plant systemic resistance, which enables moringa to fend off pests more effectively [162]. They enhance the plant’s overall health and vigor by improving nutrient uptake, which, in turn, reduces the susceptibility to pest attacks [162,163]. This enhanced nutrient status supports the activation of systemic defense mechanisms, leading to an increased production of defensive compounds such as phytoalexins and secondary metabolites that deter aphids [160,164]. Additionally, mycorrhizal associations can modify root exudate profiles, attracting natural enemies of aphids and thereby further reducing pest pressures [160].

Moreover, the benefits of AMF extend beyond pest resistance. Since pest infestations like those from aphids often lead to chlorophyll degradation and nutrient imbalances that impair photosynthesis [160], Arbuscular Mycorrhizal Fungi (AMF)-mediated improvements in plant health are critical. By promoting efficient nutrient uptake and enhancing the plant’s physiological state during stress, AMF can help maintain optimal photosynthetic rates and, ultimately, improve crop yield [160,164]. Research on *M. oleifera* cultivation consistently demonstrates that high planting density significantly influences biomass yield [165]. Mabapa et al. [9] found a positive correlation between planting density and above-ground biomass accumulation; the study revealed that the highest planting density of approximately 435,000 plants/ha yielded the greatest amount of biomass of 527–2867 kg/ha. This finding aligns with Goss [166], who reported an increased dry matter yield with the highest density, reaching a maximum of 3.4 t/ha at 197,528 plants/ha. While studies like Nouman et al. [167], Amaglo et al. [168], and Fadiyimu et al. [169] focused on harvest frequency, Amaglo et al. [168] specifically highlighted that closer plant spacing (5 × 5 cm) yielded significantly higher biomass (10–30 t/ha) compared to wider spacing (5 × 10 cm), which had a lower yield (3–12 t/ha). Interestingly, denser planting initially improved soil moisture retention, but this effect diminished over time due to increased competition for resources like light, water, and nutrients. This observation is supported by Amaglo et al. [168] and Gadzirayi et al. [170,171], as they indicated that relatively close planting spaces resulted in taller plants due to competition for growth resources. This underscores the importance of careful field management, including irrigation and fertilization, to mitigate resource competition and maximize yield in high-density *M. oleifera* cultivation.

Elevated plant population densities correspond with increased biomass production in both above-ground (shoots) and below-ground (roots) plant organs. Conversely, lower plant densities exhibit reduced root biomass, suggesting that plants in such conditions experience sufficient resource availability (water and nutrients) and do not require extensive root development for resource acquisition [172]. Increased plant density can enhance plant growth, particularly when root and shoot systems intertwine, leading to competition for resources [172]. Optimal plant densities enable efficient resource utilization and minimize inter- and intraspecific competition [173]. Plant density significantly influences crop yield, with closer spacing generally resulting in higher yields per plant [173].

A study conducted by Issaka et al. [174] revealed that high plant densities promote the development of extensive root systems, facilitating deeper soil penetration and improved nutrient and water uptake. Such root systems improve resilience to environmental stressors like drought. Increased biomass production also implies greater foliage, improving light interception and photosynthesis, ultimately boosting yields [175]. A positive correlation exists between above-ground biomass and seed yield [176].

However, exceeding optimal plant densities can trigger excessive competition, hindering productivity [177]. While high densities generally increase leaf area index and photosynthetically active radiation capture, excessively low intra-row spacing can lead to competition, etiolation, and reduced growth [177]. Therefore, high plant densities are ideal for initial seedling establishment and robust root development. Mendieta-Araica et al. [178] investigated the impact of planting density (100,000 plants/ha and 167,000 plants/ha) and nitrogen fertilization (0, 261, 521, and 782 kg N/ha) on the biomass production and chemical composition of *M. oleifera.*

Their findings revealed that a higher planting density of 167,000 plants per hectare resulted in significantly greater total dry matter yield (TDMY) and fine fraction yield (FFDM) [178]. Specifically, the higher density yielded 21.2 tons per hectare of TDMY and 19.2 tons per hectare of FFDM, compared to 11.6 tons per hectare of TDMY and 11 tons per hectare of FFDM for the lower density of 100,000 plants per hectare [176]. This difference highlights the positive correlation between planting density and biomass production within the tested range [178]. Furthermore, the study observed a faster growth rate in the higher planting density treatment. This outcome likely contributes to the increased biomass yield, as a faster growth rate allows plants to accumulate biomass more rapidly.

This claim is further supported by research by Foidl et al. [179], which assessed the impact of planting density on moringa dry matter yield and revealed a positive correlation between the two factors. Their findings indicated a substantial increase in DM yield from 5 to 44 tons per hectare as planting density rose from 350,000 to 16,000,000 plants per hectare [171]. However, the study also observed elevated mortality rates at the highest densities. Consequently, the authors concluded that a planting density of 1,000,000 plants per hectare struck an optimal balance between maximizing yield and minimizing mortality-related losses [171]. Other studies, like the one by Mabapa et al. [9], suggest a lower optimal planting density, ranging from 435,000 to 500,000 plants/ha. This discrepancy highlights the importance of considering local conditions and conducting site-specific trials to determine the most effective planting density for maximizing moringa yield.

### 7.1. Impact of Elevated Planting Density Stress on Moringa: Biochemical and Physiological Perspectives

#### 7.1.1. Biochemical and Physiological Changes

High planting density stress in moringa leads to resource competition; specifically, increased competition for light, water, and nutrients triggers physiological and morphological adaptations. Plants may elongate stems to enhance light capture, leading to thinner stems and altered biomass allocation [180]. Density stress influences phytohormone balance. Elevated levels of abscisic acid are associated with stress responses, including stomatal closure to conserve water, potentially limiting photosynthesis [181]. Conversely, cytokinins, often associated with growth promotion, may be suppressed under high-density stress [182].

Competition for resources can lead to oxidative stress, characterized by the accumulation of reactive oxygen species [183]. Moringa plants activate antioxidant defense mechanisms, producing enzymes like superoxide dismutase, catalase, and peroxidase, as well as non-enzymatic antioxidants like phenolic compounds and flavonoids, to scavenge ROS and mitigate oxidative damage [69]. Furthermore, the shading effect highly induced by neighboring plants reduces light availability, impacting photosynthesis. Moringa may exhibit reduced chlorophyll content and altered photosystem II efficiency under high-density stress. [184]. Competition for nutrients can limit nutrient uptake and assimilation. Nitrogen deficiency, common under high-density stress, can impact protein synthesis and overall growth [184].

#### 7.1.2. Impact on Growth and Development

High planting density can negatively impact plant growth and development due to increased competition for resources. Violle et al. [185] found that competition leads to a reduced growth rate, resulting in shorter plants with diminished leaf area and overall biomass. Furthermore, stress induced by high planting density can delay key phenological stages, such as flowering and seed set, ultimately impacting reproductive output [186]. Additionally, Hodge et al. [187] observed alterations in root architecture, with plants developing shallower root systems that limit their ability to access water and nutrients from deeper soil layers.

### 7.2. Phytochemical Production

The impact of density stress on phytochemical production in moringa is complex and can be influenced by various factors, including the specific compound, growth stage, and severity of stress. Some studies suggest that moderate density stress can raise the production of certain phytochemicals, potentially as a defense mechanism [188,189]. For instance, phenolic compounds, known for their antioxidant properties, may accumulate in response to oxidative stress [69]. Conversely, severe stress can negatively impact phytochemical synthesis by limiting resource availability and diverting energy toward stress response mechanisms [190].

## 8. Use of Arbuscular Mycorrhizal Fungi in Alleviating Salinity Stress and Plant Density Stress

In view of these diversified significant applications of *M. oleifera* and its impact on improved livelihoods and health, it is crucial that proper and viable agronomic practices for increased productivity of *M. oleifera* be identified and established. Identifying and selecting AMF species or strains that are well adapted to local soil conditions and tailored to the crop’s specific needs is a crucial step in optimizing plant productivity and stress resilience [191]. This process begins with screening native AMF populations to determine which fungi have naturally evolved in the local ecosystem and have already adapted to its unique climatic, soil, and environmental conditions [191]. Native strains may possess inherent traits that enable them to perform effectively under local abiotic stresses, such as soil salinity, and biotic challenges, such as competition in high-density plantings [192].

In addition to exploring local fungal diversity, the use of commercial inoculants is also a valuable strategy. These products are often formulated with specific AMF species that have been scientifically validated to enhance plant growth under various stress conditions [193]. For instance, some commercial strains are known for their ability to improve nutrient uptake, bolster plant defenses, and promote robust root systems, which are particularly beneficial in saline soils or densely planted fields. By incorporating such tailored inoculants, farmers can ensure that their crops receive the maximum benefit from AMF symbiosis, leading to enhanced growth, improved stress tolerance, and ultimately higher yields [193]. Firstly, seed coating involves applying AMF inoculants directly onto the surface of seeds, typically using a binder to adhere the fungal propagules [193]. This method ensures that the fungi are in close proximity to the emerging roots during germination, facilitating early colonization and establishment of the symbiotic relationship [193]. Seed coating is considered an effective and precise method for delivering microbial inoculants, with the potential for large-scale application in crops such as wheat, maize, and cowpea [193].

Secondly, seed priming involves hydrating seeds in a controlled manner, sometimes with additives, to initiate the early stages of germination without allowing radicle emergence. This process can improve germination rates and seedling vigor [194]. When combined with AMF inoculation, seed priming can enhance the establishment of the mycorrhizal association, leading to improved plant growth and stress tolerance [195]. Thirdly, root dipping involves immersing the roots of seedlings or transplants into a suspension containing AMF propagules before planting [195]. This technique allows for immediate contact between the fungi and plant roots, promoting rapid colonization upon transplanting. Root dipping is particularly beneficial in nursery settings or when transplanting seedlings, as it enhances the establishment of the mycorrhizal association, leading to better nutrient absorption and increased plant vigor [195]. 

Each of these inoculation techniques offers distinct advantages and can be selected based on specific crop requirements, soil conditions, and management practices. By effectively integrating AMF through these methods, farmers can enhance plant health, improve yields, and promote sustainable agricultural systems. Furthermore, mixing inoculum with soil entails thoroughly blending AMF inoculum into the soil to ensure an even distribution of fungal propagules [196]. Uniform mixing promotes consistent colonization of plant roots across the cultivated area, leading to enhanced nutrient uptake and improved plant performance [196]. To implement this method, the first step is preparation, which involves obtaining a suitable AMF inoculum, such as spores, infected root fragments, or hyphal networks [197]. Next, the inoculum must be mixed with the soil or growth medium to ensure thorough integration and even distribution of the fungal propagules [197]. Once the inoculum is well incorporated, planting can proceed by sowing seeds or transplanting seedlings into the inoculated soil, allowing the AMF to establish symbiotic relationships with the plant roots as they develop [198]. This method is particularly beneficial in controlled environments such as greenhouses or nurseries, where precise management of soil composition is feasible [198]. By ensuring widespread distribution of AMF, plants can uniformly benefit from the fungi’s ability to enhance nutrient absorption and stress tolerance.

Layering or banding is another agronomic practice that may increase the effectiveness of AMF inoculation on *M. oleifera* cultivation. It involves placing AMF inoculum at specific soil depths or in concentrated bands near the plant root zones [199]. This targeted application positions the fungal propagules strategically, facilitating efficient colonization of the roots. The process begins with trench preparation, where small trenches or furrows are created in the planting area, positioned where the plant roots will develop [200]. The AMF inoculum is then applied directly into these trenches or furrows, forming a concentrated band of fungal propagules. After application, the inoculum is lightly covered with soil to protect it and maintain optimal conditions for fungal activity [199]. Finally, seeds are sown, or transplants are placed above or adjacent to the inoculated bands, ensuring that the developing roots will encounter the AMF as they grow [199]. This method is particularly advantageous in field settings, especially for row crops, as it localizes the inoculum application to areas where it is most needed, optimizing the use of inoculum and enhancing colonization efficiency [199].

Implementing these soil inoculation techniques can significantly improve plant growth and resilience. By fostering a symbiotic relationship between AMF and plant roots, these methods enhance nutrient uptake, particularly phosphorus, and improve plant tolerance to various environmental stresses. Careful consideration of the specific crop, soil conditions, and environmental factors will guide the selection of the most appropriate inoculation method, contributing to sustainable and productive agricultural practices.

The use of Arbuscular Mycorrhizal Fungi (AMF) is one sustainable alternative practice that aims to alleviate abiotic and biotic stress factors, such as salinity stress and plant density stress, respectively, that may hamper the productivity, biomass accumulation, and the biosynthesis of secondary metabolites.

### 8.1. Arbuscular Mycorrhiza Fungi Alleviation of Soil Salinity

Soil salinization presents a critical global challenge, jeopardizing both food security and environmental sustainability [41]. Soil salinization generates excessive reactive oxygen species, which are detrimental to plant growth, making the study of salt stress and its impact on plants a crucial area of research. A recent study by Afrangan et al. [200] focused on mitigating this oxidative damage. Their findings highlighted the significant roles of *Glomus versiforme* and *Micrococcus yunnanensis* in regulating redox balance and ion homeostasis in *Brassica napus* L. crops under salt stress conditions. While salinity negatively affects plant growth by inhibiting vegetative processes and reducing net assimilation, ultimately lowering yield productivity, Arbuscular Mycorrhizal Fungi have emerged as a promising solution [201].

Numerous studies demonstrate that AMF can boost plant growth and yield under salt stress conditions. For instance, Giri et al. [202] reported that *Acacia nilotica* seedlings inoculated with AMF exhibited significantly greater root and shoot biomass compared to non-mycorrhizal seedlings. Similarly, Al-Karaki [203] observed enhanced growth parameters in mycorrhizal tomato plants, including increased shoot and root dry weight, fruit yield, individual fruit weight, and overall fruit number. This enhanced growth is often attributed to the improved nutrient acquisition facilitated by AMF, particularly phosphorus (P). Matamoros et al. [204] emphasized the crucial role of mycorrhizal associations in enhancing P uptake, with estimates suggesting that external hyphae can contribute up to 80% of a plant’s P requirements.

The beneficial effects of AMF on P acquisition are particularly pronounced under saline conditions. Giri et al. [202] demonstrated that mycorrhizal *Acacia nilotica* plants maintained significantly higher P content (1.2, 1.2, 0.9 and 0.6%) across a range of salinity levels (1.2, 4, 6.5 and 9.5 dS m^−1^) compared to their non-mycorrhizal counterparts (0.6, 0.5, 0.2 and 0.1%). This highlights the ability of AMF to mitigate the negative impacts of salinity on P uptake. Further supporting this, Shokri and Maadi [205] observed a decline in P concentration in *Trifolium alexandrium* plants with increasing salinity levels. However, mycorrhizal plants consistently exhibited higher P concentrations compared to non-mycorrhizal plants across all salinity levels, underscoring the vital role of AMF in enhancing P uptake under salt stress. The improved P nutrition in AM-inoculated plants has been linked to a range of physiological benefits, including enhanced growth rate, increased antioxidant production, and improved nodulation and nitrogen fixation in legumes Feng et al. [206].

Ait-El-Mokhtar et al. [207] emphasize the benefits of AMF symbiosis in saline environments, particularly its role in mitigating the detrimental effects of salinity on photosynthesis [208]. The interaction of AMF with medicinal plants like moringa could mitigate salt-induced stress on plant productivity, plant health, leaf area, and biomass while improving the root-to-shoot dry mass ratio [209]. These benefits are partly attributed to modifications in the fungal environment and development of extensive mycelial networks modulating water retention, absorption, soil volume, and AMF–host water relations [209]. Metabolically, these effects involve the exclusion of Na^+^, reducing toxic Na^+^ accumulation and promoting selective K^+^, facilitated by the AMF–plant symbiosis [209]. Mycorrhizal inoculation has been shown to improve the growth and resilience of various plant species, particularly under saline conditions [41]. The mechanisms by which Arbuscular Mycorrhizal Fungi confer salinity tolerance in plants are multifaceted. These include mechanisms that maintain osmotic balance, stimulate antioxidant activities to protect against damage by reactive oxygen species (ROS), increase the photosynthetic rate, and regulate hormonal levels to abate the harmful effects of salts on plant growth and development, allowing AMF to mitigate plant salinity stress. Mycorrhizal colonization can induce changes in the relative abundance of organic solutes, such as modifying the composition of carbohydrates and inducing the accumulation of specific osmolytes like proline, which facilitate osmotic adjustment [210]. For instance, inoculating *Ocimum basilicum* L. with mycorrhizal fungi significantly enhanced photosynthetic rate, gas exchange traits, chlorophyll content, and water use efficiency [211]. Similarly, a study by Borde et al. [212] demonstrated the beneficial effects of AMF inoculation on *Allium sativum* plants grown under salinity stress. These plants exhibited improved growth, particularly in terms of leaf area index and fresh and dry biomass [212].

Further research suggests that mycorrhizal inoculation under moderately saline conditions significantly increases fresh and dry weights, as well as nitrogen concentrations in both shoots and roots [213]. This growth enhancement is attributed to the ability of AMF to improve nutrient uptake and water relations in plants. Moreover, AMF inoculation has been shown to positively influence growth-promoting factor levels, particularly cytokinin concentrations [214]. Elevated cytokinin levels enhance photosynthetic flux under salt stress, further supporting plant growth [214]. The positive effects of AMF on plant growth under salt stress may also be linked to alterations in the polyamine pool, which plays a crucial role in stress tolerance [215]. Arbuscular Mycorrhizal Fungi (AMF) play a crucial role in mitigating various plant stresses, including salinity, by influencing plant physiology and enhancing stress tolerance mechanisms. One study highlighted the role of strigolactone, a plant hormone, in AMF-mediated salt stress alleviation in lettuce plants [216]. Increased strigolactone levels were found to significantly reduce the detrimental effects of salt stress on AMF-treated plants [216]. Arbuscular Mycorrhizal Fungi (AMF) also contribute to reduced oxidative stress in plants exposed to salinity [212]. They achieve this by decreasing lipid membrane peroxidation, a key indicator of oxidative damage. Furthermore, AMF enhances the accumulation of organic acids in plants under saline conditions, improving osmoregulation [41]. This effect is supported by evidence showing increased organic acid and betaine production in AMF-colonized maize plants grown in saline soil [41]. The ability of AMF to modify plant physiology extends to both morphological and physiological traits, enabling plants to better cope with stress. For instance, AMF enhance the uptake of essential minerals like phosphorus, copper, zinc, and water, contributing to improved plant survival under stress [126]. In other words, AMF are a valuable ally for plants facing various environmental challenges, including salinity, grazing, metal contamination, intraspecific competition, and drought [217]. Their ability to improve nutrient uptake, reduce oxidative stress, and improve osmoregulation makes them essential for promoting plant health and resilience in adverse conditions.

Soil salinization is a growing concern that threatens global food security and agricultural productivity, particularly in arid and semi-arid regions where yearly precipitation is not as high as evapotranspiration, posing a significant threat to agricultural productivity [218]. The cause of salinity in these regions stems from a combination of climatic, geological, and anthropogenic factors [219]. Limited precipitation coupled with high surface evaporation rates creates a hydrological imbalance, leading to salt accumulation in the soil. This effect is further compounded by the weathering of native rocks, which releases salts into the environment. Additionally, irrigation practices using saline water and inadequate agricultural management techniques contribute significantly to the escalating salinity issue [219]. Various sources estimate that approximately 1 billion hectares of land globally are affected by salinity [220,221]. Projections indicate that this number could rise to 50% of all arable land by 2050, leading to increased food insecurity [222]. This issue is particularly concerning in Sub-Saharan Africa, where 19 million hectares are already affected [223], including over 3 million hectares in Tanzania alone [224].

High salt concentrations, especially sodium and calcium ions, create a hostile environment for plant growth. These ions disrupt ion balance, damage cellular structures, impair photosynthesis and respiration, hinder protein synthesis, and lead to nutrient deficiencies [225]. There is a need, therefore, to establish how the tree will perform and adapt to local South African conditions, which are drier, warmer, and predominantly sandy soils and inherently low in nitrogen and phosphorus [226].

Research on the relationship between salt tolerance and mycorrhizal associations presents a complex picture. Otlewska et al. [227] found that while halophytic plants, known for their natural salt tolerance, generally show a low affinity for mycorrhizal fungi, these fungi can still benefit non-halophytic crops in saline environments, particularly by reducing yield losses in moderately saline soils. However, Pan et al. [228] provide evidence suggesting that AMF can indeed benefit halophytes by promoting a balance in ion and osmolyte accumulation. Specifically, they found that AMF help halophytes increase their uptake of essential inorganic ions like potassium and calcium while decreasing the accumulation of osmolytes such as proline and soluble sugars, ultimately leading to enhanced biomass production even under salinity stress [229].

Arbuscular Mycorrhizal Fungi (AMF) augment the uptake of essential nutrients, including phosphorus, nitrogen, and other growth-promoting micronutrients, compensating for the reduced nutrient availability in saline soils [229]. Arbuscular Mycorrhizal Fungi (AMF) promote root growth and branching, increasing the surface area for water and nutrient absorption. Likewise, AMF contribute to the production of osmoprotectants like polyols in plants, helping maintain cellular water balance under saline conditions [230]. By enhancing nutrient acquisition, promoting root development, and influencing plant physiological processes, AMF play a crucial role in alleviating the detrimental effects of salinity and improving plant growth and resilience in salt-affected soils.

### 8.2. Arbuscular Mycorrhiza Fungi Alleviation of Plant Density Stress

Elevated planting densities, while capable of augmenting yield, induce heightened intraspecific competition among *M. oleifera* individuals. This competition for finite resources, including water, essential nutrients, and photosynthetically active radiation (PAR), generates a stress response within the plants [231]. Consequently, growth, developmental processes, and even the biosynthesis of secondary metabolites, specifically valuable phytochemicals, are negatively impacted by intensified intraspecific competition for resources [231]. As intra-plant spacing decreases, this stress intensifies, prioritizing resource acquisition over vegetative growth and reproductive development [232]. Within this context, the symbiotic relationship between *M. oleifera* and Arbuscular Mycorrhizal Fungi becomes particularly advantageous.

Arbuscular Mycorrhizal Fungi (AMF) establish a symbiotic relationship with *M. oleifera*, effectively expanding the plant’s root system via an extensive hyphal network. This network extends beyond the rhizosphere, accessing resources spatially unavailable to the plant’s own roots [233]. This is particularly crucial for the acquisition of immobile nutrients, such as phosphorus, which are rapidly depleted in the rhizosphere under conditions of high planting density [234]. Furthermore, AMF improves nutrient uptake by actively solubilizing and mobilizing nutrients into forms more readily assimilated by the plant [235]. The interconnected hyphal network also facilitates inter-plant nutrient transfer, promoting a more homogeneous distribution of resources within the densely planted population [235].

Arbuscular Mycorrhizal Fungi (AMF) provide benefits to plants beyond nutrient uptake, such as improved water relations. The extensive hyphal network of AMF augments both the absorption and translocation of water to the host plant [236]. This enhanced water acquisition and transport confers an adaptive advantage, particularly in competitive environments [237]. Moreover, this improved water management contributes to increased drought tolerance, a crucial trait in high-density planting systems where water availability is often a limiting factor or resource [238].

Furthermore, AMF acts as a stress mediator, influencing the production of plant hormones involved in stress responses [239,240]. By modulating these hormonal signals, AMF helps the moringa plants better cope with the physiological stress brought on by crowding and resource competition [241]. Arbuscular Mycorrhizal Fungi (AMF) function as stress mediators in plants, influencing the biosynthesis and signaling pathways of phytohormones involved in stress responses [242]. By modulating these hormonal signals, AMF improves the ability of moringa plants to withstand physiological stress induced by high-density planting, such as increased competition for resources [47]. Essentially, AMF establishes a multifaceted support system that enables moringa to flourish even under the challenging conditions of high-density cultivation.

Arbuscular Mycorrhizal Fungi (AMF) have demonstrated the ability to redistribute phosphorus from high to low concentration areas, as evidenced by in vitro studies [243,244]. This nutrient reallocation through the common mycorrhizal network can influence the competitive dynamics among interconnected plants. The symmetry of competition, whether plants compete equally or benefit at the expense of another, can be affected by variations in sink strength (a plant’s ability to draw resources from the CMN) and alterations in below-ground nutrient transfer dynamics [245].

It is generally accepted that under resource-limited conditions, interspecific competition (competition between different species) is more likely than intraspecific competition (competition within the same species) due to differing resource requirements among species [238,239]. Additionally, the efficiency of different plant species interacting with the same AMF species can vary, potentially mitigating competition [246].

When a particular plant individual or species within a community exhibits superior growth and fitness compared to its neighbors, it can have cascading effects on the overall plant community structure [19]. This disparity in performance can lead to shifts in plant community dynamics, diversity, and even ecosystem productivity [247]. Over time, these differences in response to AMF-mediated interactions can result in changes to the relative abundance of plant species within the community. Therefore, AMF, through their influence on nutrient distribution and plant performance, can act as significant drivers of plant community structure and composition [19].

### 8.3. Arbuscular Mycorrhiza Fungi Influence on Phytochemical Biosynthesis

While the influence of AMF on *M. oleifera* growth and phosphorus uptake has been documented [69], research on their impact on bioactive compounds and microelements in edible moringa tissues remains limited. Arbuscular Mycorrhizal Fungi (AMF) are known to influence not only plant growth and mineral uptake but also the accumulation of bioactive compounds and microelements with potential human health benefits in various plant species [42,238,239]. For instance, studies on tomatoes (*Solanum lycopersicon*) indicate that AMF promote the safe production of high-quality food without introducing mutagenic or allergenic compounds [248,249]. Furthermore, AMF colonization has been shown to affect the accumulation of various beneficial compounds in different plant species. Other health-promoting metabolites that are positively affected by AMF are tocopherols and anthocyanins in lettuce (*Lactuca sativa*) [249,250], as well as increased accumulation of flavonoids and phenolic acids on crops, herbs, and medicinal plants.

A study conducted by Tong et al. [250] investigated the impact of both single and mixed inoculation with two AMF species on the β-carotene content in sweet potato tubers. The researchers found that inoculation with AMF generally increased β-carotene concentrations, particularly under conditions of low phosphorus supply [250]. Under low phosphorus conditions, inoculation with a single AM fungus boosted β-carotene levels by 52%, while mixed inoculation led to a 68% increase. In high phosphorus conditions, β-carotene levels rose slightly, with a 10% increase from single inoculation and a 16% rise from mixed inoculation compared to non-mycorrhizal plants [250]. The effect of AM fungi was more significant in low phosphorus environments, emphasizing their role in enhancing nutrient uptake and carotenoid production when phosphorus is scarce [250].

On the other hand, a study conducted by Carderelli et al. [251] examined the influence of AMF on the concentrations of alloins and β-polysaccharides in two aloe species (*Aloe arborescens* and *Aloe barbadensis*) under different fertilization and salinity conditions. The study demonstrated that AMF improved the concentration of aloins and β-polysaccharides in both aloe species [251]. The β-polysaccharides concentration in *A. barbadensis* was notably higher than in *A. arborescens* and increased by 33% following inoculation with AMF. Additionally, the β-polysaccharide concentration in *A. barbadensis* was 45.7% higher compared to *A arborescens*, and it rose by 33% when the plants were inoculated with *Glomus intraradices* and *Glomus mosseae* [251]. Another study conducted by Yuan et al. [252] examined the effects AMF had on the active ingredients in medicinal plants and revealed that AMF inoculation increased the contents of medicinal active ingredients by 27%, with a particularly notable increase observed in flavonoids (68%) and terpenoids (53%).

Furthermore, a study conducted by Müller et al. [253] assessed whether AMF would influence glucosinolate levels in *Tropaeolum majus* and *Carica papaya* after application of jasmonic acid. The study revealed that all mycorrhizae inoculated (+M) *Tropaeolum* plants experienced an alteration in glucosinolate biosynthesis compared to the controls [253]. Benzyl glucosinolate increased two- to four-fold, whereas 3-indolylmethyl glucosinolate was increased in the Golden Gleam and Scarlet Gleam cultivars. The Mycorrhizal inoculated (+M) roots of *T. canariense* were found to contain decreased levels of aliphatic glucosinolate, whereas the concentrations of p-hydroxybenzyl glucosinolate and p-methoxybenzyl glucosinolate increased nine-fold and more than five-fold [253], respectively.

The study conducted by Cosme et al. [47] shows that root colonization by AMF can affect the levels of important bioactive compounds and mineral elements in leaves of moringa, a vegetable crop appreciated for its nutritional and health-promoting value. Specifically, AMF increased levels of glucosinolates, had no effect on flavonoids and phenolic acids, reduced levels of carotenoids, increased Copper (Cu), and interacted positively to increase Zinc (Zn) in leaves [47]. Arbuscular Mycorrhizal Fungi (AMF) colonization did not change the total growth of moringa, which contrasts with studies showing positive effects [254,255].

## 9. Conclusions and Future Perspective

This review delved into the remarkable potential of AMF as a sustainable solution for enhancing *M. oleifera* cultivation, particularly in regions facing salinity and land constraints. A growing body of scientific literature provides compelling evidence for the positive impact of AMF on plant growth, especially under challenging environmental conditions.

When it comes to combating salinity stress, AMF act as a lifeline for moringa. They form an intricate hyphae network extending far beyond the plant’s root system, effectively increasing the surface area for absorption. This heightened exploration of the soil allows moringa to access and uptake essential nutrients like phosphorus, nitrogen, and zinc more efficiently, even when their availability is compromised by high salt concentrations. Furthermore, AMF improve the soil structure, creating a more favorable environment for water retention. This is crucial for moringa, as salinity often leads to osmotic stress, making it difficult for the plant to absorb water. Moreover, AMF bolster the plant’s natural defenses against salinity-induced oxidative stress by enhancing the production of antioxidant enzymes.

In addition to alleviating salinity stress, AMF also hold immense promise for optimizing moringa cultivation under high planting densities. As the demand for moringa and its valuable products increases, farmers are looking for ways to maximize yields from limited land areas. However, high planting densities often lead to competition for resources, negatively impacting plant growth. Arbuscular Mycorrhizal Fungi (AMF) can help overcome this challenge by increasing the overall availability of nutrients in the soil and facilitating their efficient distribution among densely planted moringa plants.

Future research should prioritize several key areas to fully harness the potential of AMF in moringa cultivation. First, identifying and characterizing moringa-specific AMF species will be crucial. Different AMF species exhibit varying compatibility and effectiveness with different plant species. Therefore, identifying those that form the most beneficial symbiotic relationships with moringa, particularly under specific stress conditions, will be essential. Second, optimizing AMF inoculation methods, including the timing, dosage, and application techniques, will be vital for maximizing their effectiveness under varying salinity levels and planting densities. Finally, conducting long-term field studies is imperative to evaluate the long-term efficacy, feasibility, and economic viability of AMF application in real-world moringa cultivation systems. By deepening our understanding of the intricate interplay between moringa and AMF, and translating this knowledge into practical applications, we can pave the way for more sustainable and resilient agricultural practices. This will increase moringa production and contribute to global food security by providing a nutritious and adaptable crop that can thrive in challenging environments. While the current research confirms the potential of AMF to improve stress resilience in *Moringa*, further studies are necessary to explore the specific mechanisms by which AMF influence secondary metabolite biosynthesis and their potential to enhance *Moringa* productivity in diverse environmental contexts. Additionally, a deeper understanding of AMF interactions with plant density and salinity, particularly through field and greenhouse trials, will help optimize their application in sustainable agricultural practices.

## Figures and Tables

**Table 1:** *Moringa oleifera* ethnomedicinal use.

Species	Part	Medicinal Use	References
*Moringa oleifera*	Gum	Fever, dysentery, asthma	[58]
	Seeds	Warts	[59]
	Oil	Acute rheumatism, Gout	[60]
	Flowers	Tumor, inflammation, hysteria,	[53]
	Roots	Toothache, anthelmintic, ant paralytic	[61]
	Bark	Stomach pain, ulcer, poor vision, joint pain, hypertension, anemia, diabetes, uterine disorders, impaired vision, joint pain, diabetes, anemia, hypertension, toothaches, hemorrhoids, and various stomach ailments, including ulcers and digestive problems	[53,62]
	Leaves	Malaria, arthritis, hypertension, diabetes, swelling, stomach pain, common cold, elicit lactation	[63]

## Data Availability

No new data were created or analyzed in this study.
